# A Comparative Study on the Health Status and Behavioral Lifestyle of Centenarians and Non-centenarians in Zhejiang Province, China—A Cross-Sectional Study

**DOI:** 10.3389/fpubh.2019.00344

**Published:** 2019-11-22

**Authors:** Chao Rong, Shu-Hua Shen, Lu-Wei Xiao, Qi Huang, Han-Ti Lu, Hong-Xian Wang, Zheng-Xiang Li, Xiao-Ming Wang

**Affiliations:** ^1^School of Humanities and Management, Zhejiang Chinese Medical University, Hangzhou, China; ^2^Bloomberg School of Public Health, Johns Hopkins University, Baltimore, MD, United States; ^3^The First Affiliated Hospital of Zhejiang Chinese Medical University, Hangzhou, China; ^4^Yiwu Hospital of Traditional Chinese Medicine, Yiwu, China; ^5^Wenling Hospital of Traditional Chinese Medicine, Wenling, China

**Keywords:** centenarians, longevity, health preservation, traditional Chinese medicine, disease prevention

## Abstract

**Background:** The growth rate of centenarians was unusually rapid in recent decades, ushering in an era of longevity. This study aims to explore the difference between centenarians and non-centenarians using quantitative research, and to scientifically guide residents to develop the correct lifestyle and health care ways.

**Methods:** From October 2013 to August 2017. A cross-sectional survey was conducted on 271 centenarians and 570 non-centenarians by using a questionnaire to assess longevity and health issues which was developed for the needs of the study, who came from 29 counties and districts in 11 cities of Zhejiang province, China. Two hundred and fifty-five valid questionnaires were returned, with an effective response rate of 94.1%. Meanwhile, data of 526 non-centenarians from Zhejiang province was collected as a control group, with an effective response rate of 92.3%.

**Results:** The prevalence rates of tumor, stomach and duodenal ulcer, diabetes, bronchial asthma, and chronic obstructive pulmonary disease, tuberculosis among centenarians were all lower than those among non-centenarians. The oral health of centenarians is better than that of non-centenarians. The consumption of coarse cereals, pasta, other staple foods and fruits among centenarians was higher than that of non-centenarians. The percentage of centenarians who smoke or engage in recreational activities every day was lower than that of non-centenarians.

**Conclusions:** We should give full play to the role of preventive medicine and health management to safeguard the health of residents. Pay attention to oral health, and develop the good habit of loving teeth. The diet should be rich and varied, and increase the intake of grains and fruits. Give up smoking, limit alcohol, spirit-preserving with calming, follow the law of scientific regimen.

## Introduction

Health and longevity have been human dreams since ancient times. With the continuous growth of the economy and the stability and development of society, human life expectancy is increasing. By the end of 2015, China's life expectancy had reached 76.2 years, with 73.2 years for men and 79.9 years for women. Life expectancy in Zhejiang province is 80.5 years, with 77.7 years for men and 83.8 years for women. Given that life expectancy has risen, the number of centenarians is further increasing (1).

The population division of the United Nations department of economic and social affairs released global population by age group in 2015, and it said there are 451,000 centenarians worldwide. There were 355,000 women and 96,000 men, and the number of women was significantly higher than that of men. It will rise to 1.245 million by 2030 and 3.67 million by 2050. Japan has 61,568 centenarians, and it is forecast to reach 272,000 by 2050. Britain had 13,350 centenarians in 2012. Australia is expected to have 12,001 centenarians by 2020 and 50,000 by 2050. The number of centenarians in China has increased year by year (2).

In 1990, 2000 and 2010, there were 6,681, 17,877, and 35,934 centenarians in China, respectively. The average annual growth rate exceeded 10%. And, from 1953 to 2001, it increased more than 4-fold in half a century. The growth rate was unusually rapid, ushering in an era of longevity (3). The number of centenarians in China was 58,789 in 2014, and Chinese centenarians were mostly women, accounting for three-quarters of the total, of which 70% lived in rural areas. By the end of 2016, there were 2,307 centenarians in Zhejiang province, which is much higher than the life expectancy in Zhejiang province. The United Nations has designated 7.5 centenarians per 100, 000 people as the “land of longevity,” showing the scarcity of this special population.

A paper by Braveman et al. (4) named The Social Determinants of Health: Coming of Age, and described the rapidly growing studies on the social determinants of health in the United States and elsewhere, and highlighted health is affected not only by living and working conditions, but also by more upstream determinants, such as the economic and social resources and opportunities, which influence an individual's access to health-promoting living and working conditions (4). She also explained in detail how neighborhood conditions and working conditions and education and income and race and stress affect health (4). Maness and Branscum illustrated the concept of social determinants of health is widely used in health promotion and public health efforts. The World Health Organization (WHO) defined social determinants of health as “the conditions in which people are born, grow, live, work and age circumstances that are affected by the distribution of power, money and resources at local, national and global levels.” Social determinants of health problems are the main causes of health inequities, leading to inequitable health disparities within and between countries that could have been avoided. WHO created the Commission on Social Determinants of Health in 2005, which pushed forward the development of the theory, and further clarified the relationship between societal factors and health outcomes (5).

Centenarians are a special group that gains much attention and admiration of family members, relatives, friends, neighbors, and service providers (6). There are biological, psychological and social reasons for the longevity of centenarians, including genetic reasons, external environment, and behavioral habits. Most of the existing research has been done from a genetic or biological perspective (7). Some studies have been carried out from the perspective of social medicine. However, most of them are qualitative research or quantitative research with a small sample size without a control group. We aimed to explore the difference between centenarians and non-centenarians using quantitative research with large sample size and a control group, and to scientifically guide residents to develop correct lifestyle and health care methods from the psychological and social perspective (8).

## Materials and Methods

### Study Population

From October 2013 to August 2017, the methods of stratified sampling and representative sampling were adopted by the research team. First, according to administrative divisions, Zhejiang Province is divided into 11 cities. They are Hangzhou, Wenzhou, Ningbo, Jiaxing, Taizhou, Jinhua, Shaoxing, Zhoushan, Quzhou, Huzhou, and Lishui. Second, according to the geographical location, one of the districts and counties in the east (middle), and west regions were selected in each city. In addition, we considered the representative of characteristics other than geographic location. For example, economy, occupation, education, lifestyle, climate, etc. If some districts and counties are special in these characteristics, then choose them. We finally chose a total of 29 districts and counties. A field survey was conducted on 271 centenarians by using a questionnaire to assess longevity and health issues which was developed for the needs of the study. The centenarians or families who know them best or caregivers were investigated in their homes by the investigator who had received prior standardized training. The exclusion criteria were the centenarians with no clear consciousness or with a long illness in bed or refuse to be investigated. The personal basic information of each subject is recorded in detail, which including age, gender, marriage, the spectrum of disease, diet habits, smoking, drinking, hobbies, characteristics, loneliness and medical behaviors, etc. Two hundred and fifty-five questionnaires were collected, with an response rate of 94.1%. Meanwhile, data of 526 non-centenarians (a total of 570 non-centenarians was investigated, with an response rate of 92.3%) from Zhejiang province was collected as a control group using the same sampling methods, inclusion and exclusion criteria. All of the respondents were informed of the study and signed a letter of consent.

### Measurements

#### Social Demographic Variables

Social demographic variables were collected in the following format: gender (male, female), marital status (having spouses; having no spouse, it includes single, divorced, and widow), and the age recorded was consistent with their ID card.

#### Diseases

The centenarian and/or families and/or caregiver were directly asked if they had ever been diagnosed by a doctor with any of the following: hypertension, stroke, stroke sequelae, tumor, coronary heart disease, stomach and duodenal ulcer, diabetes, bronchial asthma, chronic obstructive pulmonary disease, and tuberculosis. Results were recorded as either presence or absence of the disease or not sure. Dental and visual status variables were collected in the following format: dental status [almost perfect teeth (32 teeth), retained more than 50% of their teeth (16 or more teeth), <50% of their teeth (<16 teeth, and >0 teeth), all teeth removed (0 teeth)], the use of denture (use, not use), visual status (good vision, moderate vision, poor vision, extremely poor vision).

#### Diet Habits

Diet habits variables were collected in the following format: diet preferences (vegetarians, meat and vegetable eaters, meat eaters). The respondents were asked whether they ate the following foods: meat, fish and other aquatic products, eggs, tea, bean products, rice, cereals, pasta, other staple foods, fruits. Results were recorded as either eat or not eat or sometimes eat.

#### Smoking, Drinking, and Hobbies

Smoking, drinking, and hobbies variables were collected in the following format: smoking (yes, no), drinking (yes, no), engage in recreational activities (every day, sometimes, basically not), watch TV or listen to radio, read books and newspapers (often, not frequently, not).

#### Character, Loneliness, and Medical Behaviors

Character, loneliness, and medical behaviors variables were collected in the following format: character type (introverted, extroverted, others), loneliness (always, often, occasionally, never, not aware of), satisfaction of their current lives (Satisfied, relatively satisfied, dissatisfied, very dissatisfied, not aware of), timely medical treatment when they were ill (can, can not).

### Data Analysis

Epidata3.1 software was used for data entry and spss21.0 software for data statistics and analysis. The mean, standard deviation, median, quartile spacing, minimum value, and maximum value for continuous variables were described, as well as the frequency and percentages for nominal variables. The Chi-square test was used to analyze the prevalence and lifestyle differences between centenarians and non-centenarians. The continuous variables did not show a normal trend through the Shapiro-Wilk test, however, in addition to using the median and quartile for describing the age in the paper, the mean and standard deviation was increased to facilitate their interpretation and comparison with other populations.

### Ethical Approval

The Medical Ethics Committee of First Affiliated Hospital of Zhejiang Chinese Medical University granted ethical approval. All participants were informed of the intent and requirements of the study and asked to sign the informed consent form without any perceived pressure or inducement. All participants were guaranteed their right to refuse to engage in or to withdraw from this study at any time.

## Results

### Basic Social Demographic Characteristics

The average age of 255 centenarians was 102, the standard deviation was 2, the median age was 102, quartile spacing was 101–103, the minimum age was 100, the maximum age was 115. The average age of 526 non-centenarians was 89, the standard deviation was 6, the median age was 89, quartile spacing was 84–94, minimum age 78, maximum age 99. Among the centenarians surveyed, 83 were males, accounting for 32.5%, and 172 were females, accounting for 67.5%. Among non-centenarians, 259 were males, accounting for 49.2%, and 267 were females, accounting for 50.8%. Using the chi-square test, the chi-square value is 19.437 and the *P*-value <0.001. It is showed that the proportion of women in centenarians is higher than that of non-centenarians, and the difference was statistically significant. Consistent with WHO statistics on the global population, women live longer than men. Among the centenarians, 24 people were living with spouses, accounting for 9.6%. Two hundred and twenty-seven people were not living with a spouse, accounting for 90.4%. missing 4 people data. Among non-centenarians, 130 people were living with spouses, accounting for 24.7%. Three hundred and ninety-six people were not living with a spouse, accounting for 75.3%. Using the chi-square test, the chi-square value is 24.551 and the *P*-value <0.001. Among the centenarians, 250 people had been married, accounting for 99.6%. One person had not been married, accounting for 0.4%. Among non-centenarians, 520 people had been married, accounting for 98.9%. Six people had not been married, accounting for 1.1%. The percentage of centenarians who not living with a spouse was higher than that of non-centenarians, and the difference is statistically significant. It can be seen that the percentage of couples who can spend their centenarians together is relatively low. As people age, most people die near the age of life expectancy.

### Spectrum of Disease

The prevalence rates of tumor, stomach and duodenal ulcer, diabetes, bronchial asthma, and chronic obstructive pulmonary disease, tuberculosis among centenarians were 0.8, 1.2, 1.6, 4.7, 0.0%, respectively, and those among non-centenarians were 4.9, 6.8, 6.7, 13.9, 3.8%, respectively. The chi-square test showed that the former was lower than the latter, and the difference was statistically significant. It is showed that the prevalence rates of tumor, stomach and duodenal ulcer, diabetes, bronchial asthma, and chronic obstructive pulmonary disease, tuberculosis among centenarians were all lower than those among non-centenarians. The prevalence rate of hypertension among centenarians was significantly higher and that of non-centenarians (Table 1).

**Table 1:** A comparative analysis of the prevalence of centenarians and non-centenarians.

		**Centenarians**	**Non-centenarians**	**Chi-square**	***P***
Hypertension	No	168 (65.9%)	384 (73.0%)	4.203	0.044
	Yes or not sure	87 (34.1%)	142 (27.0%)		
Stroke, Stroke sequelae	No	242 (94.9%)	508 (96.6%)	1.266	0.328
	Yes or not sure	13 (5.1%)	18 (3.4%)		
Tumor	No	253 (99.2%)	500 (95.1%)	8.593	0.002
	Yes or not sure	2 (0.8%)	26 (4.9%)		
Coronary heart disease	No	231 (90.6%)	464 (88.2%)	0.989	0.393
	Yes or not sure	24 (9.4%)	62 (11.8%)		
Stomach and duodenal ulcer	No	252 (98.8%)	490 (93.2%)	11.628	<0.001
	Yes or not sure	3 (1.2%)	36 (6.8%)		
Diabetes	No	251 (98.4%)	491 (93.3%)	9.362	0.001
	Yes or not sure	4 (1.6%)	35 (6.7%)		
Bronchial asthma and chronic	No	243 (95.3%)	453 (86.1%)	14.898	<0.001
Obstructive pulmonary disease	Yes or not sure	12 (4.7%)	73 (13.9%)		
Tuberculosis	No	255 (100.0%)	506 (96.2%)	9.951	<0.001
	Yes or not sure	0 (0.0%)	20 (3.8%)		

*Because the number of people choosing not to know was small, they were classified as the number of people who were ill*.

Among centenarians, 9 people had almost perfect teeth (32 teeth), accounting for 4.5%. Eighteen people retained more than 50% of their teeth (16 or more teeth), accounting for 9.0%. There were 66 people with <50% of their teeth (<16 teeth, and >0 teeth), accounting for 33.2%, and 106 people with all teeth removed (0 teeth), accounting for 53.3%, and 56 people have missing data. Those among non-centenarians were 1 (0.2%), 39 (7.4%) 278 (52.9%), 208 (39.5%), respectively. Using the chi-square test, the chi-square value was 38.206, and the *P*-value <0.001.

The number of centenarians who used denture was 53, accounting for 20.8%, and 202 people who did not use it, accounting for 79.2%, and those among non-centenarians were 160 (30.7%), 361 (69.3%), respectively. Using the chi-square test, the chi-square value was 8.470, and the *P*-value was 0.004. The percentage of centenarians using denture was lower than that of non-centenarians, and the difference was statistically significant. It can be seen that the oral health of centenarians is better than that of non-centenarians.

Among centenarians, 128 had good vision, accounting for 51.0%, 62 had a moderate vision, accounting for 24.7%, 26 had poor vision, accounting for 10.4%, and 35 had extremely poor vision, accounting for 13.9%, and 4 people have missing data. Those among non-centenarians were 395 (75.5%), 51 (9.8%), 69 (13.2%), 8 (1.5%), respectively, and 3 people have missing data. Using the chi-square test, the chi-square value was 89.228, and the *P*-value <0.001. The visual condition of centenarians was worse than that of non-centenarians, and the difference was statistically significant.

### Diet Habits

The diet preferences for meat and vegetable combination of centenarians was higher than that of non-centenarians, and the diet preferences for vegetarian of centenarians was lower than that of non-centenarians, the difference was statistically significant. The consumption of meat, fish and other aquatic products, eggs, tea, bean products among centenarians was lower than that of non-centenarians, and the difference was statistically significant. The consumption of coarse cereals, pasta, other staple foods and fruits among centenarians was higher than that of non-centenarians, and the difference was statistically significant (Table 2).

**Table 2:** A comparative analysis of diet types between centenarians and non-centenarians.

		**Centenarians**	**Non-centenarians**	**Chi-square**	***P***
Meat	Eat	205 (82.0%)	513 (100.0%)	98.127	<0.001
	Not eat	45 (18.0%)	0 (0.0%)		
Fish and other aquatic products	Eat	100 (40.0%)	516 (100.0%)	384.99	<0.001
	Not eat	150 (60.0%)	0 (0.0%)		
Eggs	Eat	77 (30.8%)	514 (100.0%)	459.806	<0.001
	Not eat	173 (69.2%)	0 (0.0%)		
Tea	Eat	35 (14.1%)	285 (56.5%)	123.137	<0.001
	Not eat	214 (85.9%)	219 (43.5%)		
Bean products	Eat	10 (4.0%)	439 (84.3%)	446.285	<0.001
	Not eat	239 (96.0%)	82 (15.7%)		
Rice	Eat	242 (97.6%)	519 (98.7%)	1.209	0.368
	Not eat	6 (2.4%)	7 (1.3%)		
Coarse cereals	Eat	21 (8.5%)	1 (0.2%)	41.817	<0.001
	Not eat	227 (91.5%)	525 (99.8%)		
Pasta	Eat	54 (21.8%)	6 (1.1%)	100.340	<0.001
	Not eat	194 (78.2%)	520 (98.9%)		
Other staple foods	Eat	16 (6.5%)	0 (0.0%)	34.652	<0.001
	Not eat	232 (93.5%)	526 (100.0%)		
Fruits	Eat	65 (26.4%)	57 (10.9%)	41.054	<0.001
	Sometimes eat	154 (62.6%)	339 (64.6%)		
	Not eat	27 (11.0%)	129 (24.6%)		

*The missing data was not included*.

### Smoking, Drinking, and Hobbies

The percentage of centenarians who smoke was lower than that of non-centenarians. Non-smoking has been shown to promote healthy longevity. The percentage of centenarians who engage in recreational activities every day was lower than that of non-centenarians. The percentage of centenarians who often watch TV or listen to the radio was lower than that of non-centenarians. It is showed that as older people age, they become less interested in outside entertainment (Table 3).

**Table 3:** A comparative analysis of smoking, drinking, and hobbies between centenarians and non-centenarians.

		**Centenarians**	**Non-centenarians**	**Chi-square**	***P***
Smoking	Yes	34 (13.9%)	111 (21.1%)	5.714	0.017
	No	211 (86.1%)	415 (78.9%)		
Drinking	Yes	58 (23.9%)	144 (27.4%)	1.056	0.333
	No	185 (76.1%)	382 (72.6%)		
Engage in recreational activities	Everyday	12 (5.2%)	82 (15.6%)	15.753	0.000
	Sometimes	95 (41.5%)	196 (37.3%)		
	Basically not	122 (53.3%)	247 (47.0%)		
Watch TV or listen to radio	Often	31 (12.6%)	110 (20.9%)	8.387	0.015
	Not frequently	99 (40.2%)	178 (33.8%)		
	Not	116 (47.2%)	238 (45.2%)		
Read books and newspapers	Often	18 (7.4%)	42 (8.0%)	5.375	0.068
	Not frequently	30 (12.3%)	38 (7.2%)		
	Not	195 (80.2%)	445 (84.8%)		

*The missing data was not included*.

### Character, Loneliness, and Medical Behaviors

The percentage of centenarians who were introverted was higher than that of non-centenarians. The percentage of centenarians who were never lonely was higher than that of non-centenarians. The percentage of centenarians who were able to receive timely medical treatment when they were ill was lower than that of non-centenarians (Table 4).

**Table 4:** A comparative analysis of character, loneliness, and medical behaviors of centenarians and non-centenarians.

		**Centenarians**	**Non-centenarians**	**Chi-square**	***P***
Character type	Introverted	60 (24.5%)	35 (6.7%)	49.475	<0.001
	Extroverted	140 (57.1%)	381 (72.4%)		
	Others	45 (18.4%)	110 (20.9%)		
Loneliness	Always	2 (0.8%)	7 (1.3%)	190.772	<0.001
	Often	18 (7.3%)	60 (11.4%)		
	Occasionally	87 (35.4%)	395 (75.1%)		
	Never	109 (44.3%)	29 (5.5%)		
	Not aware of	30 (12.2%)	35 (6.7%)		
Satisfaction with your current life	Satisfied	150 (64.7%)	360 (68.4%)	3.147	0.533
	Relatively satisfied	65 (28.0%)	120 (22.8%)		
	Dissatisfied	7 (3.0%)	22 (4.2%)		
	Very dissatisfied	1 (0.4%)	1 (0.2%)		
	Not aware of	9 (3.9%)	23 (4.4%)		
Timely medical treatment	Can	156 (62.7%)	445 (94.1%)	115.510	<0.001
	Can not	93 (37.3%)	28 (5.9%)		

*The missing data was not included*.

## Discussion

We examined the differences of the health status and behavioral lifestyle between centenarians and non-centenarians in Zhejiang province, China, and aims to guide residents to develop the correct lifestyle and health care ways. The chi-square test showed that there may be a correlation between the longevity of centenarians and their better health. The oral health of centenarians is better than that of non-centenarians. The visual condition of centenarians was worse than that of non-centenarians. The consumption of coarse cereals, pasta, other staple foods and fruits among centenarians was higher than that of non-centenarians. As older people age, they become less interested in outside entertainment. The percentage of centenarians who were never lonely was higher than that of non-centenarians. The percentage of centenarians who were able to receive timely medical treatment when they were ill was lower than that of non-centenarians.

This study found that the proportion of women (67.5%) in centenarians was much higher than that of men (32.5%), while in non-centenarians, the proportion of men and women was not much different. This is consistent with Newman and Brach‘s findings (9). Possible reasons are as follows: The mortality rates for many diseases are higher in men than in women. For example, heart disease, stroke, cancer, motor vehicle accident, chronic lung disease (9). Men's overweight rate, smoking rate, and other unhealthy lifestyles are higher than women (9). Men have more accidents and less use preventive health care services than women (9). This study found that the prevalence rates of tumor, stomach and duodenal ulcer, diabetes, bronchial asthma, chronic obstructive pulmonary disease and tuberculosis in centenarians were lower than that of non-centenarians, and the difference was statistically significant. The results of this study are consistent with those of Pedro and Ailshire. Pedro et al. pointed out that centenarians have a high self-perception of health, low frequency of diabetes, dyslipidemia, cardiovascular disease (1). Ailshire, Beltran-Sanchez, and Crimmins found that centenarians are generally healthier than non-surviving members of their cohort, and several centenarians with no self-reported diseases or functional impairments. About 23% of centenarians with no major chronic disease and ~18% had no disability. 55% of centenarians without cognitive impairment (10). The previous study have identified the several important aspects of socioeconomic indicators for longevity: higher overall economic development level, public expenditure on health, and so on (11, 12). da Silva et al. confirmed that the low-frequency consumption of red meat, cholesterol, and heme iron may be one of the keys to longevity for centenarians (13). Stathakos et al. implied that it is possible to achieve longevity while the elder still being relatively healthy, autonomous and socially active (14). Deng et al. found that socioeconomic factors such as local infrastructure, health care facilities, and economic status might be the most important contributors to the longevity of people aged 60–90 years in Guangxi, China (15).

This study suggests that there may be a correlation between the longevity of centenarians and their better health. Therefore, it is very necessary to maintain the good health of residents. The family doctor contract service currently being implemented in China, and use the “Internet plus” method to regularly collect data on residents' health conditions, carry out a health risk assessment and health intervention. We should give full play to the role of preventive medicine and health management to safeguard the health of residents.

This study found that the tooth retention of centenarians was better than that of non-centenarians, and the proportion of centenarians using dentures was lower than that of non-centenarians, with statistically significant differences. It can be seen that the oral health of centenarians is better than that of non-centenarians, and oral health is related to spleen and stomach and the absorption of nutrients. Tan, Peres, and Peres found that the retention of teeth is associated with better oral health-related quality of life (16). It was observed by Shiue that adults reported ever hypertension, depression, diabetes, poor mental health status, and poor self-rated health were dissatisfied with their current teeth appearance (17). Oral health is an important part of human health. WHO regards oral health as one of the ten signs of human health. Therefore, we should pay attention to oral health, and develop the good habit of loving teeth.

Those who are centenarians have a meat and vegetable collocation diet habit are higher than those who are non-centenarians. The consumption of meat, fish and other aquatic products, eggs, and soy products among centenarians is lower than that of non-centenarians. Centenarians eat more coarse cereals, pasta, other staples and fruits than non-centenarians. The above differences were statistically significant. Accordingly, the edible of flesh and fish should not be overmuch, meat and vegetable collocation are advisable. The study by Robert and Fulop found that nutritional factors play an important role in longevity, in addition, unhealthy nutrition combined with a marked sedentary life is a major cause of obesity, reaching up to 30% of our population. Obesity is a serious risk factor for many diseases (18). The diet should be rich and varied, and increase the intake of grains and fruits.

The percentage of centenarians who smoke is lower than that of non-centenarians, and the percentage who watch TV and radio regularly every day is lower than that of non-centenarians. It is proved again that non-smoking promotes health and longevity. The study by Robert and Fulop also found that smoking and alcohol are the most important risk factors for longevity (18). Willig et al. also proposed the correct use of medicines and having physical, mental and spiritual health are considered by the oldest-old as essential for health preservation (19). This study found the percentage of centenarians who were introverted was higher than that of non-centenarians. This view is consistent with Law et al. who pointed out that Australian centenarians were currently low in openness and extraversion and high in neuroticism (20). However, Martin et al. had a different view, and showed that Georgia centenarians had several unique traits: low levels of neuroticism, high competence, and high Extraversion. The reason for the difference may be that the subjects come from different countries (21). Davey indicated that centenarians had the following characteristics; impulsiveness, higher self-consciousness, and deliberation, but lower ideas, compliance, and self-discipline (22).

The study also shows that as people get older, their interest in outside entertainment diminishes. The percentage of centenarians who are introverted and never feel lonely is higher than that of non-centenarians. People should respond to the lifestyle of smoking cessation and alcohol restriction and mentality balance advocated by WHO. Meanwhile, follow the regimen thoughts of traditional Chinese medicine (TCM). One of four classics of Chinese medicine–Huang Di Nei Jing (Huangdi's Internal Classic) has a classic discussion on regimen: People of the ancient time who knew how to cultivate health, regulate yin and yang, conform to the ways of health preservation, control feed and drinks, live regularity and not to work absurdly, is thus in the state of somatic and spiritual harmony, and could fulfill their natural span of life of 100 years. Experts of health preservation in successive dynasties pay lots of attention to the close relationship between quiescence based vitality preserving and health. They lay stress on the thought of “spirit-preserving with the calming pattern.” In the regimen in TCM, “keeping the mind in the interior” is the base of disease prevention and treatment as well as the core of mind cultivation. It mainly refers to self-control and regulation of man's consciousness, thoughts, and mental state to keep the harmony between the human body and environment. We suggested that give up smoking, limit alcohol, spirit-preserving with calming, follow the law of scientific regimen.

This study had some limitations. Firstly, only Zhejiang province was selected as a sample province to survey this research program, which does not represent the whole longevity situation of China. As this study was restricted to Zhejiang province only, it may not apply to international populations either, given the specificities of the Chinese human species. Secondly, this study only focused on longevity, but the quality of life of the centenarians was not investigated. Thirdly, there are other factors associated with longevity that were not investigated, such as educational level, economic status, living situation, and these factors are worth further study. Fourth, this study was a cross-sectional one, and such type of studies are only indicative of differences between segments of the population and cannot provide definite answers regarding the causes of longevity.

## Conclusions

We should give full play to the role of preventive medicine and health management to safeguard the health of residents. Pay attention to oral health, and develop the good habit of loving teeth. The diet should be rich and varied, and increase the intake of grains and fruits. Give up smoking, limit alcohol, spirit-preserving with calming; follow the law of scientific regimen. This study has important guiding significance for advocating people to form healthy lifestyles. Using the international universal scale to measure the quality of life of centenarians is worthy of further research in the future.

## Data Availability Statement

The datasets generated for this study are available on request to the corresponding author.

## Ethics Statement

The studies involving human participants were reviewed and approved by the Ethics Committee of the First Affiliated Hospital of Zhejiang Chinese Medical University. The patients/participants provided their written informed consent to participate in this study. Written informed consent was obtained from the individual(s) for the publication of any potentially identifiable images or data included in this article.

## Author Contributions

L-WX, QH, and X-MW designed the present study. S-HS, H-XW, and Z-XL assisted in the literature reviews and investigation. The data analysis and original draft were conducted by CR and H-TL. All authors contributed to and have approved the final manuscript.

### Conflict of Interest

The authors declare that the research was conducted in the absence of any commercial or financial relationships that could be construed as a potential conflict of interest.
